# SARS-CoV-2: An Update on Potential Antivirals in Light of SARS-CoV Antiviral Drug Discoveries

**DOI:** 10.3390/vaccines8020335

**Published:** 2020-06-23

**Authors:** Hatem A. Elshabrawy

**Affiliations:** Department of Molecular and Cellular Biology, College of Osteopathic Medicine, Sam Houston State University, Conroe, TX 77304, USA; hatem.elshabrawy@shsu.edu

**Keywords:** 2019-nCoV, SARS-CoV, SARS-CoV-2, COVID-19, antivirals, antibodies

## Abstract

Coronaviruses (CoVs) are a group of RNA viruses that are associated with different diseases in animals, birds, and humans. Human CoVs (HCoVs) have long been known to be the causative agents of mild respiratory illnesses. However, two HCoVs associated with severe respiratory diseases are Severe Acute Respiratory Syndrome-CoV (SARS-CoV) and Middle East Respiratory Syndrome-CoV (MERS-CoV). Both viruses resulted in hundreds of deaths after spreading to several countries. Most recently, SARS-CoV-2 has emerged as the third HCoV causing severe respiratory distress syndrome and viral pneumonia (known as COVID-19) in patients from Wuhan, China, in December 2019. Soon after its discovery, SARS-CoV-2 spread to all countries, resulting in millions of cases and thousands of deaths. Since the emergence of SARS-CoV, many research groups have dedicated their resources to discovering effective antivirals that can treat such life-threatening infections. The rapid spread and high fatality rate of SARS-CoV-2 necessitate the quick discovery of effective antivirals to control this outbreak. Since SARS-CoV-2 shares 79% sequence identity with SARS-CoV, several anti-SARS-CoV drugs have shown promise in limiting SARS-CoV-2 replication in vitro and in vivo. In this review, we discuss antivirals described for SARS-CoV and provide an update on therapeutic strategies and antivirals against SARS-CoV-2. The control of the current outbreak will strongly depend on the discovery of effective and safe anti-SARS-CoV-2 drugs.

## 1. Introduction

Coronaviruses (CoVs) are a group of positive single-stranded (_+_ss) RNA viruses that belong to the family *Coronaviridae* [1]. Different CoVs have been isolated from multiple species of mammals and birds [2]. Based on the genome sequence and the animal species they infect, CoVs have been classified into four genera: *Alphacoronavirus*, *Betacoronavirus*, *Gammacoronavirus*, and *Deltacoronavirus* [1]. Alphacoronaviruses and betacoronaviruses are known to exist in mammals, whereas gamma and deltacoronaviruses circulate in birds and mammals [2]. The first human CoVs (HCoVs) were discovered in the 1960s and to date, seven HCoVs are known to cause respiratory diseases with varying severity [3,4]. Four HCoVs (HCoV-OC43, HCoV-HKU1, HCoV-229E, and HCoV-NL63) cause 15% of common colds with mild symptoms, whereas three viruses cause severe respiratory diseases with viral pneumonia [3,4]. These three HCoVs include Severe Acute Respiratory Syndrome-CoV (SARS-CoV), Middle East Respiratory Syndrome-CoV (MERS-CoV), and the most recently described SARS-CoV-2 [3,4]. SARS-CoV-2 causes the COVID-19 disease, which has resulted in thousands of deaths to date [5]. SARS-CoV-2 was first isolated from critically ill patients in December 2019 [4]. These patients were connected to the Huanan Seafood Market in Wuhan, China [4]. Similar to SARS-CoV, bats have been suggested as the primary host of SARS-CoV-2; however, an intermediate host is yet to be identified [6,7]. Before the discovery of SARS-CoV, there was no urgency in the development of anti-CoV therapeutics. However, the high fatality rate in SARS-CoV, MERS-CoV, and SARS-CoV-2 outbreaks necessitates the development of effective antivirals. Many research groups have developed antivirals against SARS-CoV (Table 1). Similarly, research has been underway to identify antivirals that are effective against SARS-CoV-2 (Table 2). Genetic sequencing has shown that SARS-CoV-2 shares 79% identity with SARS-CoV [7]. Based on this finding, efforts to discover antiviral drugs against SARS-CoV-2 have been guided by our understanding of SARS-CoV and the discovery of several anti-SARS-CoV drugs. In this review, we will describe the antiviral drugs showing efficacy against SARS-CoV and will highlight the antiviral drugs that have been reported to be effective against SARS-CoV-2 in vitro and in vivo. We will also include clinical trials that are underway to prove the efficacy and safety of such antivirals.

## 2. SARS-CoV

**Figure 1:**
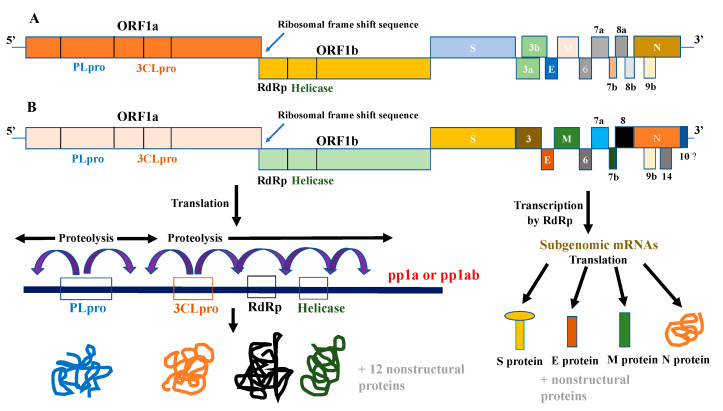
Genomic organization of (**A**) Severe Acute Respiratory Syndrome-coronavirus (SARS-CoV) and (**B**) SARS-CoV-2. The structure of SARS-CoV and SARS-CoV-2 genomes includes ORF1a and ORF1b occupying two-thirds of the genomes at the 5′ end. ORF1a and ORF1b are translated through a ribosomal frame shift sequence into two polyproteins 1a and 1b (pp1a and pp1ab) which are processed by 3CLpro and PLpro proteases to produce nonstructural proteins, including RdRp, that are important for viral replication. The other one-third of the genomes is comprised of ORFs that code for structural (S, M, E, and N) and nonstructural proteins. ORF: open reading frame; RdRp: RNA-dependent RNA polymerase; 3CLpro: picornavirus 3-chymotrypsin-like protease; PLpro: papain-like protease 2.

The SARS-CoV outbreak started in Guangdong, China in 2002–2003 and resulted in 8098 cases and 774 deaths [8]. Based on the genome sequence, SARS-CoV was classified as a betacoronavirus [7,9]. Similar to other CoVs, SARS-CoV has a _+_ssRNA genome that is 30 kb in size [10]. The 5′ end of this genome has a cap structure, whereas the 3′ end is polyadenylated. The genome is bound to nucleocapsid (N) protein, forming the core of the viral particle [10]. In addition to N protein, SARS-CoV has three other structural proteins that are inserted in the viral envelope and are named spike (S), membrane (M), and envelope (E) proteins [10,11]. The S protein is a 1255-amino-acid homotrimeric glycoprotein that projects from the surface of the viral particles [10,12]. Each monomer is composed of two domains: S1 and S2. SARS-CoV infects unciliated bronchial epithelial cells and type II pneumocytes after binding to its receptor, angiotensin-converting enzyme 2 (ACE2) [13]. The S1 domain of the S protein mediates the binding of SARS-CoV to the ACE2 receptor [14]. After binding of SARS-CoV to ACE2, the S protein is cleaved by the plasma membrane-bound serine protease TMPRSS2 or by cathepsin L in late endosomes [15,16,17,18,19]. The cleavage of S protein exposes the fusion peptide of the S2 domain which mediates the fusion of the viral envelope with cellular membranes, allowing the viral RNA to enter the cytoplasm. Once viral RNA is in the cytoplasm, the 5′ overlapping open reading frames 1a and 1b (ORF 1a and ORF 1b) are translated into two polyproteins pp1a and pp1ab [10,20] (Figure 1A). pp1a and pp1ab are then processed by papain-like protease 2 (PLpro) and a picornavirus 3-chymotrypsin-like protease (3CLpro) into 16 mature nonstructural proteins (nsps) that are important in viral life cycle, including RNA-dependent RNA polymerase (RdRp) [20,21] (Figure 1A). RdRp uses the _+_ssRNA viral genome as a template to generate multiple copies of viral genome and to produce subgenomic mRNAs [21]. Subgenomic mRNAs are translated to produce the structural (S, M, E, and N) and nonstructural viral proteins (Figure 1A). Once sufficient structural proteins and viral genomes are formed, assembly of new viral particles starts [21]. The N protein binds to viral genomes to form the nucleocapsid core of the viral particles. The N protein of the nucleocapsid interacts with M protein that is embedded, together with the S and E protein, in the membranes of the endoplasmic reticulum (ER)–Golgi intermediate compartment (ERGIC). These interactions result in assembly and budding of new viral particles into the Golgi compartment and they finally exit the cell via exocytosis. The M and E proteins are believed to mediate the assembly and release of mature viral particles.

Since the 2002–2003 outbreak, several research groups have focused on identifying and developing antivirals that can inhibit SARS-CoV infection. These antivirals include monoclonal antibodies and small molecules that target different steps in the viral life cycle (Table 1). Potential drug candidates that inhibited SARS-CoV by targeting viral proteins (direct-acting antivirals) or host cell proteins (host-directed antivirals) are classified below based on their mechanism of action.

## 3. SARS-CoV Antivirals

### 3.1. Entry Inhibitors (Antibodies, Recombinant Proteins, Peptides, and Small Molecule Antivirals)

#### 3.1.1. Antibodies

The humoral response to SARS-CoV was studied in 20 patients during the 2003 outbreak [22]. Viral-specific IgM and IgG were absent in the patient sera for 1 week after symptom onset. At week 2, 16 patients had IgM in their sera, whereas 17 had IgG. At week 3, all patients had IgG and high levels persisted for three months after symptom onset. The IgM levels remained high until week 4, started to decline over time, and eventually disappeared at week 12. None of the healthy volunteers in the study were positive for viral-specific IgM or IgG. These results suggest that SARS-CoV-specific IgG antibody responses exist and that antibodies persist for at least 3 months. The previous findings further suggested that humoral immune responses may offer protection against SARS-CoV. Soon after the previous study, one group expressed and functionally characterized SARS-CoV S protein [12]. They identified the receptor-binding domain (RBD) within the S1 domain of S protein (amino acids residues 303 to 537). Another group demonstrated that the RBD was a 193-amino acid fragment (residues 318–510) within the S1 domain and that it bound ACE2 more efficiently than the full S1 domain (residues 12–672) [14]. There was an immediate need for therapies that could inhibit SARS-CoV infection during this outbreak. Passive transfer of sera from mice that were previously infected with SARS-CoV protected naïve mice from SARS-CoV intranasal challenge [23]. Furthermore, recombinant protein fragments derived from different regions of SARS-CoV S protein were capable of eliciting humoral immune responses in animals, and the immune sera neutralized SARS-CoV infection in vitro and in vivo [24,25,26,27,28,29]. Anti-SARS-CoV S protein-neutralizing antibodies were detected in sera of SARS-CoV patients, and convalescent plasma obtained from recovered patients was capable of treating patients with active SARS-CoV infections [30,31,32,33]. The previous findings suggested that S protein induces the production of highly potent neutralizing antibodies in infected patients and immunized animals. Since SARS-CoV S protein binds to ACE2 and mediates viral entry, it is not surprising that S protein-specific antibodies would be effective in neutralizing SARS-CoV and could be used as therapeutics for active SARS-CoV infections. Several groups developed and characterized S protein-specific monoclonal antibodies that bound to either S1 or S2 domain. A large number of these antibodies were able to neutralize SARS-CoV infections in vitro and in animal models, by different mechanisms [34,35,36,37,38,39,40,41,42,43,44,45,46,47,48].

#### 3.1.2. S Protein-Binding Molecules

##### A. Lectins

Lectins are carbohydrate-binding proteins that attach to soluble carbohydrates or carbohydrate residues on surface of glycoproteins [49]. They mediate multiple biological processes and may serve as receptors for pathogens including viruses. Based on their binding characteristics, lectins have been described as antivirals that target viral glycoproteins and inhibit viral entry [50]. Griffithsin is a lectin that was isolated from the red alga *Griffithsia spp.* and showed anti-SARS-CoV activity [51]. Griffithsin inhibited SARS-CoV entry into Vero E6 cells by binding to carbohydrate residues on SARS-CoV S protein and blocking its attachment to ACE2. Griffithsin also protected mice from SARS-CoV infection. Another plant-derived lectin, *Urtica dioica* agglutinin (UDA), was tested for antiviral activity against SARS-CoV. Similar to griffithsin, UDA inhibited viral entry into Vero 76 cells and protected mice against lethal SARS-CoV challenge [52].

##### B. Emodin

**Figure 2:**
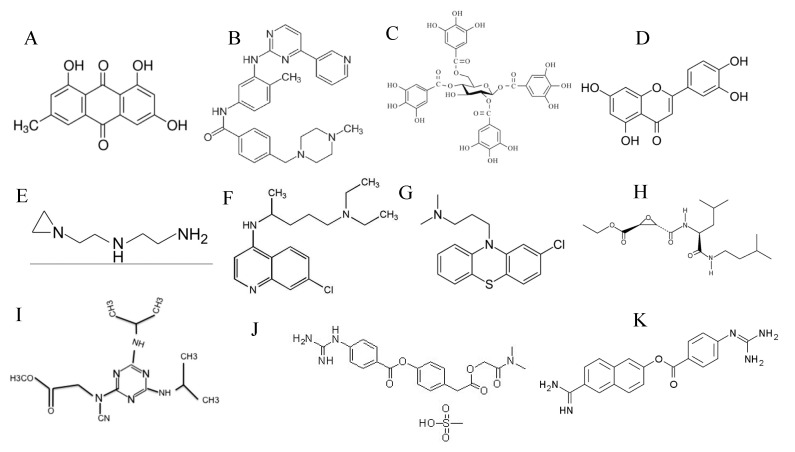
Chemical structures of SARS-CoV and SARS-CoV-2 viral entry inhibitors: (**A**) emodin, (**B**) imatinib, (**C**) tetra-O-galloyl-beta-D-glucose (TGG), (**D**) luteolin, (**E**) N-(2-aminoethyl)-1-aziridineethanamine (NAAE), (**F**) chloroquine, (**G**) chlorpromazine, (**H**) E-64D, (**I**) 5705213, (**J**) camostat mesylate, and (**K**) nafamostat.

Several groups have attempted to identify plant-derived antiviral compounds. Screening of a large number of Chinese herbs identified emodin as an anthraquinone compound (Figure 2A) that inhibited SARS-CoV S-pseudotyped viral entry into Vero E6 cells [53]. Emodin exerted its antiviral activity by blocking the interaction between S protein and ACE2, and also by inhibiting SARS-CoV 3a protein ion channel activity [53,54].

#### 3.1.3. Fusion Inhibitors

Several groups have described drugs that target the S2 domain of SARS-CoV S protein. These drugs work by inhibiting the fusogenic function of S2 domain, thus blocking viral entry. Peptides, derived from sequences in the heptad repeat 1 and 2 (HR1 and HR2) regions of the S2 domain, were synthesized and tested for the ability to inhibit SARS-CoV infection of Vero E6 cells [55]. CP1 peptide derived from HR2 inhibited SARS-CoV infection of Vero E6 cells, whereas other tested peptides did not. The study showed that CP-1 inhibited S2 fusogenic function by binding to a region on HR1. Consistent with the previous study, another group demonstrated that synthetic peptides derived from the S2 domain were able to inhibit in vitro SARS-CoV infection [56]. The anticancer drug imatinib (an Abelson (Abl) kinase inhibitor) (Figure 2B), inhibited SARS-CoV infection of Vero E6 and Calu-3 cells by blocking the fusion of SARS-CoV envelope with cell membranes [57]. Similarly, tetra-O-galloyl-beta-D-glucose (TGG) and luteolin (Figure 2C,D), were identified as inhibitors of SARS-CoV by binding to S2 domain and blocking fusion [58].

#### 3.1.4. ACE2 Inhibitors

Although most studies have focused on targeting S protein, one study aimed to block ACE2 on target cells. The small molecule N-(2-aminoethyl)-1-aziridineethanamine (NAAE) (Figure 2E) was tested as an inhibitor for ACE2 enzymatic activity and for the ability to inhibit SARS-CoV S-mediated cell to cell fusion. NAAE inhibited ACE2 activity and blocked cell–cell fusion, suggesting that it can be used as a SARS-CoV cell entry inhibitor [59].

#### 3.1.5. ACE2-Derived Peptides and Soluble Recombinant ACE2

One study has identified charged amino acids between residues 22 and 57 of ACE2 receptors as critical residues for binding to SARS-CoV S protein [60]. In this study, preincubation of SARS-CoV S-pseudotyped viral particles with synthetic peptides, comprised of the amino acids spanning the 22–57 region of ACE2, inhibited SARS-CoV entry into ACE2-expressing HeLa cells. In another study, soluble ACE2 ectodomain inhibited SARS-CoV S-pseudotyped viral entry into target cells [61]. The previous findings suggest that soluble ACE2 or ACE2 derived peptides are effective in inhibiting SARS-CoV infection and could be further developed to treat SARS-CoV patients.

#### 3.1.6. Inhibitors of Endosomal Acidification, Endocytosis, Cathepsin L, and TMPRSS2

Several compounds showed inhibitory activity against SARS-CoV either by increasing pH of the endosome, interfering with endocytosis, or inhibiting cathepsin L. Chloroquine (Figure 2F), and particularly the less toxic derivative hydroxychloroquine have long been used to treat malaria and autoimmune diseases such as rheumatoid arthritis (RA) and systemic lupus erythematosis (SLE) [62]. When tested against SARS-CoV, chloroquine inhibited the SARS-CoV infection of Vero E6 cells [63,64,65]. Chloroquine raises the pH of the endosome and thus inhibits the cleavage of SARS-CoV S by cathepsin L. Chloroquine also interfered with the glycosylation of ACE2 receptor, an effect that could impair the attachment of SARS-CoV particles to ACE2 [63,64,65]. A library of 348 FDA-approved drugs, including chlorpromazine (an antipsychotic medication, Figure 2G) and chloroquine, was tested for antiviral activity against SARS-CoV [64]. Chlorpromazine as well as chloroquine inhibited replication of SARS-CoV in Vero E6 cells. The study showed that chlorpromazine inhibited entry of SARS-CoV by blocking clathrin-mediated endocytosis.

Several groups developed and tested small molecule inhibitors of cathepsin L as potential inhibitors of SARS-CoV. All these molecules, including E-64D and 5705213 (Figure 2H,I), inhibited either live SARS-CoV or SARS-CoV S-pseudotyped viral infection in vitro and in vivo [18,66,67,68,69]. Camostat mesylate (Figure 2J) is a TMPRSS2 inhibitor that is used in Japan to treat chronic pancreatitis [18]. When tested against SARS-CoV, camostat mesylate inhibited SARS-CoV replication in vitro and in vivo [18,69]. Therefore, TMPRSS2 inhibitors and cathepsin L inhibitors are potential drugs that can be used to treat SARS-CoV infections in future outbreaks, either alone or preferably in combinations.

In addition to the above antiviral small molecules, one group tested 11 peptides that are derived from mouse β-defensin-4 for inhibiting several respiratory viruses including SARS-CoV in vitro and in vivo [70]. A short peptide named P9 blocked infection by influenza viruses, SARS-CoV, and MERS-CoV [70]. The study showed that the P9 peptide was able to bind to viral glycoproteins on the surface of SARS-CoV. Once bound to viral particle, it was endocytosed with the viral particle and inhibited endosomal acidification in late endosomes [70]. This resulted in entrapment of the viral particle in the late endosome and inhibition of the viral life cycle.

### 3.2. Protease Inhibitors

The processing of pp1a and pp1ab polyproteins by 3CLpro and PLpro generates 16 mature nonstructural proteins (nsps) including RdRp (nsp12), 3CLpro (nsp5), PLpro (nsp3), and a helicase enzyme (nsp13) [20]. Therefore, it is evident that the cleavage of polyproteins is crucial for the SARS-CoV life cycle, and blockade of viral proteases 3CLpro and PLpro could inhibit SARS-CoV replication.

#### 3.2.1. 3CLpro Inhibitors

**Figure 3:**
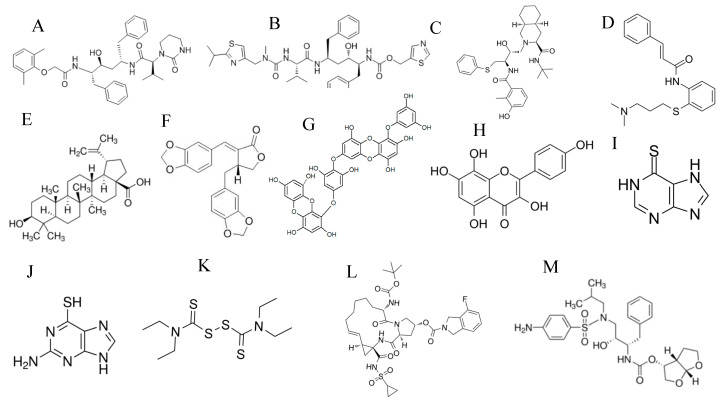
Chemical structures of protease inhibitors: (**A**) lopinavir, (**B**) ritonavir, (**C**) nelfinavir, (**D**) cinanserin, (**E**) betulinic acid, (**F**) savinin, (**G**) dieckol, (**H**) herbacetin, (**I**) 6-mercaptopurine, (**J**) 6-thioguanine, (**K**) disulfiram, (**L**) danoprevir, and (**M**) darunavir.

A Chinese group suggested the use of HIV medications (highly active antiretroviral therapy; HAART) for the management of SARS-CoV infections [71,72]. This suggestion was based on the observation that none of HIV/AIDS patients in a hospital in China contracted the SARS disease. These HIV/AIDS patients had close contact with 95 SARS patients in the same hospital and most of them were under treatment with HAART. In line with the previous observation, a research group showed that HIV-1 protease inhibitors such as the lopinavir/ritonavir (Figure 3A,B) combination were effective in SARS-CoV patients [73]. Another clinical study by a Hong Kong group reported that the use of lopinavir/ritonavir for treatment of SARS-CoV patients led to lower mortality rate [74]. Moreover, nelfinavir (an HIV-1 protease inhibitor, Figure 3C) blocked SARS CoV infection of Vero E6 cells [75].

Despite the efficacy of HIV-1 protease inhibitors against SARS-CoV in vitro and in vivo, the mechanism of action remains unclear. Inhibition of 3CLpro is a possible mechanism [76]. In addition, HIV-1 protease inhibitors were shown to have antiviral effect and antiapoptotic effect by blocking the cellular enzymes, calpains, which have been shown to be important for viral replication and viral-induced apoptosis (cytopathic effect) [77]. Other effects include interference with proteasome activity, antigen processing, and presentation, and reduction of proinflammatory cytokine production [78,79,80]. All the above effects contribute to the positive clinical outcome seen in SARS-CoV patients with the use of HIV-1 protease inhibitors.

In addition to the HIV-1 protease inhibitors, cinanserin (a serotonin antagonist that was previously tested in humans; Figure 3D) inhibited 3CLpro and SARS-CoV replication in vitro [81]. Screening of marine natural products identified a coumarin derivative, esculetin-4-carboxylic acid ethyl ester, as a novel 3CLpro inhibitor that blocked SARS-CoV replication in vitro [82]. Plant-derived compounds named betulinic acid (a sesquiterpenoid, Figure 3E) and savinin (a lignoid, Figure 3F) inhibited SARS-CoV replication by acting as competitive inhibitors of 3CL protease [83]. Quinone-methide triterpenes isolated from *Tripterygium regelii* [84], dieckol (a phlorotannin, Figure 3G) isolated from brown algae *Ecklonia cava* [85], alkylated chalcones from *Angelica keiskei* [86], and flavonoids such as herbacetin (Figure 3H), rhoifolin, and pectolinarin showed inhibition of 3CLpro in SARS-CoV [87]. Pyrazolone compounds were also described as 3CLpro inhibitors [88].

#### 3.2.2. PLpro Inhibitors

PLpro inhibitors such as diarylheptanoids from *Alnus japonica* and geranylated flavonoids from *Paulownia* tree showed antiviral activity against SARS-CoV [89,90,91,92,93]. 6-mercaptopurine (6-MP, Figure 3I), 6-thioguanine (6-TG, Figure 3J), and disulfiram (a drug used to treat alcohol dependence, Figure 3K) blocked SARS-CoV replication by inhibiting PLpro [94,95].

#### 3.2.3. Cellular Protease Inhibitors

In addition to developing inhibitors for viral proteases, other groups developed inhibitors for cellular proteases (which are involved in viral replication and virus-induced cytopathic effects) in an attempt to block SARS-CoV infection. Calpains are intracellular calcium-dependent cysteine proteases that are activated by intracellular calcium and inhibited by the endogenous molecule calpastatin [96,97]. It is believed that calpains are part of the cellular proteolytic system and are involved in different processes of cytoskeleton remodeling and apoptosis [98]. Several studies have shown that calpains play an important role in viral replication and in activation of virus-induced apoptosis [99,100]. In line with this, a research group has shown that Z-Val-Phe-Ala-CHO (calpain inhibitor III) and Val-Leu-CHO (calpain inhibitor VI) effectively inhibited in vitro SARS-CoV replication [101].

### 3.3. Viral Helicase Inhibitors

**Figure 4:**
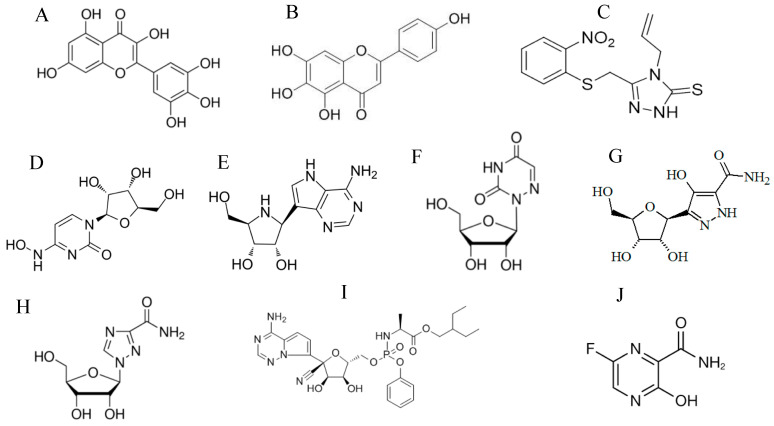
Chemical structures of SARS-CoV and SARS-CoV-2 helicase and RdRp inhibitors: (**A**) myricetin, (**B**) scutellarein, (**C**) SSYA10-001, (**D**) β-D-N4-hydroxycitidine, (**E**) galidesivir, (**F**) 6-azauridine, (**G**) pyrazofurin, (**H**) ribavirin, (**I**) remdesivir, and (**J**) favipiravir.

During replication of SARS-CoV, helicase enzyme (nsp13) catalyzes the unwinding of double-stranded viral RNA that is formed during viral replication [20]. The process involves an ATP-dependent reaction and is critical for successful viral replication [20]. A study showed that a class of compounds named bananins, which includes bananin, iodobananin, vanillinbananin, ansabananin, eubananin, and adeninobananin, inhibited the replication of SARS-CoV in cell culture by inhibiting the helicase ATPase activity [102]. Bismuth complexes inhibited SARS-CoV helicase unwinding and ATPase activities of helicase [103]. Myricetin (Figure 4A) and scutellarein (Figure 4B) were also found to inhibit the SARS-CoV by inhibiting helicase ATPase [104]. A small molecule named SSYA10-001 (triazole compound, Figure 4C) inhibited in vitro replication of SARS-CoV by inhibiting the unwinding activity of helicase enzyme but not the ATPase [105]. Despite being effective in inhibiting SARS-CoV replication, most helicase inhibitors are toxic due to inhibition of cellular ATPases or kinases, limiting their further development as SARS-CoV antivirals.

### 3.4. Replication and Transcription Inhibitors

The RdRp of SARS-CoV forms a replicase complex with other viral proteins that is crucial for viral RNA replication and transcription [20]. Analysis of the catalytic domain of SARS CoV RdRp led to the prediction of the nucleoside analogs 2′-C-methyladenosine and 2′-O-methylcitidine as potential RdRp inhibitors [106]. A research group demonstrated that another nucleoside analog, β-D-N4-hydroxycitidine (Figure 4D), blocked SARS CoV replication by inhibiting RdRp [101]. Galidesivir (BCX4430 or immucillin-A, Figure 4E) was recently developed and described as a nucleoside analog that inhibited replication of Ebola, Marburg, and other viruses in different infection models by premature termination of RNA chain synthesis [107,108,109,110]. In another study, the two nucleoside analogs 6-azauridine (Figure 4F) and pyrazofurin (Figure 4G) inhibited replication of SARS-CoV in Vero cells by inhibiting orotidine monophosphate decarboxylase and thus blocking de novo pyrimidine biosynthesis [111].

Ribavirin (Figure 4H) is another nucleoside analog that has been used as an antiviral for the treatment of many viral infections such as hepatitis C virus (HCV) and respiratory syncytial virus (RSV) [112]. It is activated intracellularly into the active form by cellular kinases and incorporates into the newly synthesized RNA chain, resulting in viral lethal mutations [112,113]. It is also believed to inhibit cellular inosine monophosphate (IMP) dehydrogenase, resulting in depletion of intracellular GTP which leads to inhibition of mRNA cap synthesis [114].

Several groups have demonstrated that ribavirin did not inhibit SARS-CoV replication in Vero cells at concentrations comparable to its in vivo concentrations [111,115,116]. However, other groups showed that ribavirin effectively inhibited SARS-CoV replication in fetal rhesus kidney cells (fRHK-4) [117]. Moreover, ribavirin was shown to inhibit SARS-CoV replication in other kidney cell lines and cancer cells [118]. Despite effectiveness of ribavirin against SARS-CoV in some in vitro studies, several clinical studies reported the ineffectiveness of ribavirin in SARS-CoV patients [119,120,121]. Furthermore, it has been shown that the viral load increased in BALB/c mice that were treated with ribavirin and other IMP dehydrogenase inhibitors, indicating that these drugs should not be considered for treating human SARS-CoV infections [122]. Moreover, the multiple side effects of ribavirin, including anemia, hypocalcemia, and hypomagnesemia discouraged its use for SARS-CoV [123,124]. The poor clinical outcome and resistance of SARS-CoV to ribavirin has been related to the specific nature of RdRp and the 3′ to 5′ exonuclease activity of nsp14, which is believed to perform proofreading of the newly synthesized RNA strand during viral replication [125].

Remdesivir (Figure 4I) is a nucleotide analog that was developed by Gilead Sciences and tested against Ebola virus in collaboration with United States Army Medical Research Institute of Infectious Diseases (USAMRIID). The drug showed efficacy against the Ebola virus in vitro, preclinically, and clinically [126,127,128]. Remdesivir inhibits Ebola RNA replication by incorporation into the newly synthesized RNA, causing a premature chain termination [127]. In addition to its activity against Ebola virus, remdesivir inhibited the replication of SARS-CoV and other coronaviruses in multiple cell types [128]. Furthermore, remdesivir reduced the viral load and mitigated disease symptoms in mice that were challenged with SARS-CoV [128]. Unlike ribavirin, SARS-CoV is not resistant to remdesivir because the drug is not removed from the growing RNA chain by the proofreading mechanism of viral exonuclease (nsp14) [128].

### 3.5. Miscellaneous Agents

N, M, and E are SARS-CoV structural proteins are essential for viral replication and assembly together with nonstructural proteins [11]. Some of these proteins modulate the host immune response to viral infections by inhibiting cellular responses such as interferon induction [129]. It has been shown that targeting viral subgenomic mRNAs for SARS-CoV E, M, N, ORF3a, ORF7a, or ORF7b genes by siRNA inhibited SARS-CoV replication in vitro [130,131]. Despite the efficacy of siRNAs in inhibiting viral infections as well as treating other diseases, clinical applications are not feasible due to the difficulties in siRNA delivery inside cells.

**Figure 5:**
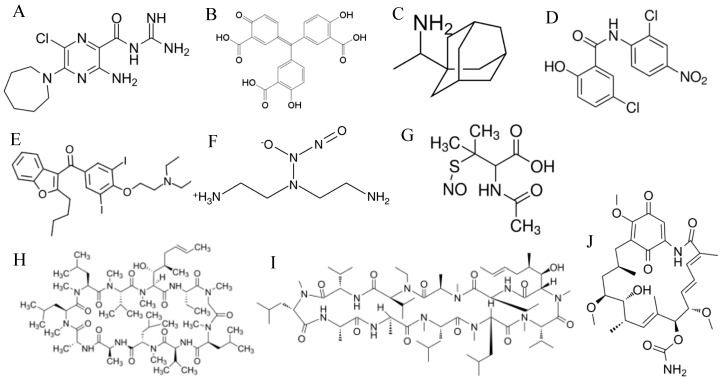
Chemical structures of miscellaneous drugs against SARS-CoV and SARS-CoV-2: (**A**) hexmethylene amiloride, (**B**) aurintricarboxylic acid, (**C**) rimantadine, (**D**) niclosamide, (**E**) amiodarone, (**F**) DETA NONOate, (**G**) S-nitroso-N-acetylpenicillamine (SNAP), (**H**) cyclosporine A, (**I**) alisporivir, and (**J**) geldanamycin.

Several drugs that are known for different therapeutic activities were tested for their antiviral activity against SARS-CoV. Hexamethylene amiloride (Figure 5A), an inhibitor of the HIV-1 Vpu ion channel, inhibited SARS-CoV replication by blocking E protein cationic-selective ion channel activity [132]. Another anti-HIV drug, aurintricarboxilic acid (Figure 5B), showed antiviral activity against SARS-CoV [133]. Inhibition of SARS-CoV attachment to target cells as well as inhibition of viral replication are the suggested anti-SARS-CoV mechanisms of aurintricarboxilic acid [133]. The anti-influenza medication rimantadine (Figure 5C), which blocks M2 ion channel of influenza virus, inhibited the replication of SARS-CoV, probably by a mechanism similar to hexamethylene amiloride [115]. Antiparasitic drugs such as niclosamide (Figure 5D), which is used to treat tapeworm infestations, inhibited replication of SARS-CoV in Vero E6 cells [134]. The anti-arrhythmic drug amiodarone (Figure 5E) blocked SARS-CoV infection of Vero cells [135]. Amiodarone was suggested to inhibit a post-entry step in SARS-CoV life cycle [135].

SARS-CoV, like other CoVs, is known to form double-stranded (ds) RNA intermediates and induce and replicate within double-membrane vesicles (DMVs) [136]. Different research groups have developed anti-CoV drugs that inhibit viral replication by either blocking formation of DMVs or inducing apoptosis in cells that contain such ds viral RNA. K22 is compound that exhibited antiviral activity against many CoVs, including SARS-CoV, by inhibiting the induction of DMVs [137]. Another protein drug known as dsRNA-activated caspase oligomerizer (DRACO) induces apoptosis in cells that are infected with CoVs by recognizing viral dsRNA intermediates using its viral dsRNA-binding domain [138]. DRACO has shown antiviral activity against many RNA viruses in vitro and in vivo. Similar to siRNAs, efficient delivery of DRACO into cells remains a challenge.

Nitric oxide (NO) is another molecule that has been considered for the treatment of SARS-CoV. NO is an effector molecule for several biological processes including vasodilation, breakdown of microorganisms in phagolysosome as part of innate immune system, inflammatory processes, and apoptosis [139]. NO possesses antiviral activity against many viruses such as herpesviruses, poliovirus, and murine coronavirus [140,141,142]. 2,2′-(hydroxynitrosihydrazino)bis-ethamine (DETA NONOate, Figure 5F) inhibited SARS-CoV in Vero and Caco2 cells. DETA NONOate dissociates in a pH-dependent manner to produce NO [111]. Another NO donor, S-nitroso-N-acetylpenicillamine (SNAP, Figure 5G), inhibited SARS-CoV replication [143]. A clinical trial in China reported the positive effect of NO on SARS-CoV patients which could be due to its vasodilation and antiviral effects [144]. These data suggest that NO could be further considered in clinical trials as an antiviral for SARS-CoV.

Although most of the antiviral drugs target viral genes or gene products, there are number of drugs that showed anti-SARS-CoV activity by inhibiting cellular proteins that are important for viral replication or by activating cellular signaling pathways that inhibit viral replication. The calcineurin pathway has been shown to be involved in SARS-CoV replication and pathogenicity [145]. During a T-cell immune response, activation of calcineurin pathway leads to activation of T cells [146]. The immunosuppressive drug cyclosporine A (Figure 5H) complexes with cellular cyclophilins and inhibits calcineurin, which leads to inactivation of T cells [147]. It was previously shown that SARS-CoV nsp1 interacted with cyclophilins, which implies that cyclophilins may serve an important role in viral replication [145]. Moreover, nsp1 interaction with cyclophilins modulated the calcineurin pathway, which enhanced activation of T cells [145]. The same study showed that cyclosporine A inhibited the replication of SARS-CoV and that of other CoVs. However, cyclosporine may not be suitable for treatment of SARS-CoV infections due to its immunosuppressive effects. Another study showed that alisporivir (Figure 5I), a non-immunosuppressive cyclosporin A-analog, inhibited in vitro replication of SARS-CoV and other CoVs [148]. This suggests that non-immunosuppressive cyclosporines may be considered for treatment of SARS-CoV.

Other drugs that inhibited SARS-CoV replication by interfering with cellular pathways or proteins include geldanamycin. Geldanamycin (Figure 5J) is an antibiotic isolated from the bacteria *Streptomyces hygroscopicus* [149]. Geldanamycin works by binding to and inhibiting heat shock protein 90 (Hsp90) which is the main mechanism for its antiviral and antitumor properties [149]. Aglycon derivatives of the antibiotics vancomycin, eremomycin, and teicoplanin also showed antiviral activities against SARS-CoV [150].

Normally when a host cell is infected with a virus, the cell senses the viral RNA and interferon expression is induced [151]. Once expressed and secreted outside the cell, interferon protects cells by limiting viral replication through the induction of expression of multiple antiviral interferon stimulated genes (ISGs) [151]. ISG protein products inhibit viral replication by multiple mechanisms which involve viral RNA degradation, inhibition of protein synthesis, and viral assembly. That is why treatment with recombinant interferon became a very common therapeutic strategy for treating viral infections such as HCV [152]. Research has shown that interferon alpha and interferon beta inhibited the in vitro and in vivo replication of SARS-CoV [153,154]. SARS patients have been treated with interferon alfa or interferon beta or its combinations with ribavirin and/or lopinavir–ritonavir. However, the outcomes of such treatments remain inconsistent [155].

**Figure 6:**
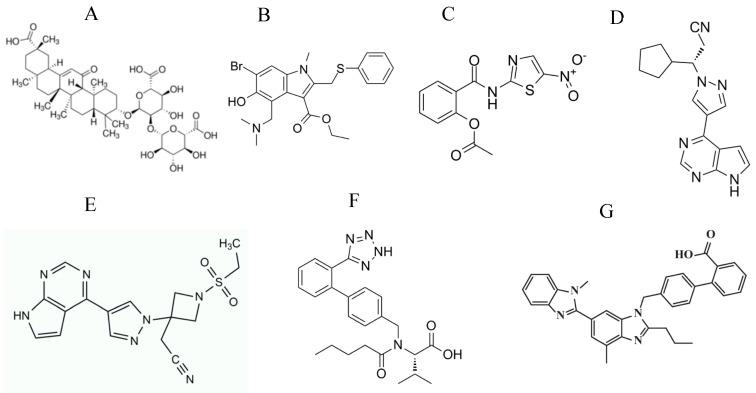
Chemical structures of miscellaneous drugs against SARS-CoV and SARS-CoV-2: (**A**) glycyrrhizin, (**B**) umifenovir, (**C**) nitazoxanide, (**D**) ruxolitinib, (**E**) baricitinib, (**F**) valsartan, and (**G**) telmisartan.

Glycyrrhizin (Figure 6A), is a triterpenoid saponin glycoside that constitutes that main bioactive component of licorice root [156]. Glycyrrhizin and its aglycone, 18β-glycyrrhetinic acid, possess anti-tumor, anti-inflammatory, and antiviral effects [157]. Glycyrrhizin exhibits antiviral activity against many viruses such as herpesviruses [158,159], flaviviruses [160], HIV-1 [161], and SARS-CoV [111,115]. Glycyrrhizin’s mechanism of action as an antiviral is not well understood. However, it is documented that glycyrrhizin interferes with several signaling proteins and transcription factors [162,163,164]. It is also possible that glycyrrhizin inhibits adsorption of SARS-CoV on target cells [111]. Glycyrrhizin may also inhibit viral replication by induction of NO production [165]. However, inhibition of SARS-CoV replication in vitro required high concentration of glycyrrhizin (100–400μM) which would be impractical to achieve in vivo if used for treating SARS-CoV patients [111,115]. It is noteworthy to mention that glycyrrhizin use should be monitored due to side effects such as increasing blood pressure and hypokalemia [157].

In addition to glycyrrhizin, active ingredients that are extracted from traditional Chinese medicinal herbs showed antiviral effects against SARS-CoV when tested in fRHK4 cells and Vero cells. This included baicalin (a flavonoid derived from *Scutellaria baicalensis*) [115], ginsenosides (steroid glycosides and triterpene saponins isolated from *Panax ginseng*) [166], aescin (saponins isolated from horse chestnut) [166], reserpine (alkaloid isolated from *Rauvolfia serpentina*) [166], and lycorine (an alkaloid isolated from the herb *Lycoris radiata*) [167].

## 4. SARS-CoV-2

SARS-CoV-2 has been identified as the causative agent of the ongoing COVID-19 pandemic that started in Wuhan, China in December 2019 [4]. Millions of cases have been reported to date, with thousands of deaths in most countries worldwide [168]. Analysis of genome sequence of SARS-CoV-2 indicates that it belongs to the betacoronaviruses, a genera which includes SARS-CoV and MERS-CoV [7]. Furthermore, genetic analyses showed that SARS-CoV-2 shared 88% sequence identity with two bat SARS-like-CoVs [7], 96% identity with a SARS-like CoV (RaTG13) [169,170], 79% identity with SARS-CoV, and only 50% identity with MERS-CoV [7]. The previous findings suggest that SARS-CoV-2 may have originated in bats, which is consistent with some reports [171,172]. The first cases of SARS-CoV-2 infections have been linked to the Huanan seafood wholesale market in Wuhan, China, which suggests that an intermediate host may exist for this virus [4]. Human-to-human transmission has been confirmed and it occurs primarily via respiratory droplets from infected individuals [7].

Patients with other underlying health conditions such as cardiovascular diseases and diabetes are at higher risk of dying from COVID-19 [173]. The incubation period for the disease ranges from 2 to 14 days and patients die in 6 to 41 days after showing symptoms [174]. The most common symptoms of COVID-19 are fever, cough, fatigue, headache, hemoptysis, diarrhea, dyspnea, and lymphopenia [4,173]. In many cases, CT scans show bilateral ground-glass opacities in lungs due to severe inflammation [175].

Similar to SARS-CoV and other CoVs, the genome of SARS-CoV-2 is a _+_ssRNA which has a cap at the 5′ end and a poly-A tail at the 3’ end [7,176]. The 5′ end of the genome is translated into two polyproteins, pp1a and pp1ab, which are processed by 3CLpro and PLpro into 16 nonstructural proteins (nsps), including RdRp (Figure 1B). The rest of the genome consists of genes coding for structural and nonstructural proteins [176] (Figure 1B). Following genome replication, the structural proteins; S, M, E, and N assemble with the viral RNA to form the viral particle [20,176]. Lung epithelial cells are the primary target of the SARS-CoV-2, and replication of virus in these cells is the cause of most of symptoms and clinical features observed in COVID-19 patients [175]. SARS-CoV-2 uses its RBD within S1 domain of S protein to bind ACE2 and infect lung cells [177]. Studies have shown that the RBD of SARS-CoV-2 S protein is similar in structure to that of SARS-CoV [7,177]. However, differences in the amino acid sequences of S proteins of both viruses and key residue substitutions within SARS-CoV-2 RBD have been reported [7,177]. These amino acid substitutions in SARS-CoV-2 RBD resulted in the higher affinity and stronger binding of SARS-CoV-2 S protein to ACE2 than SARS-CoV S protein [178,179,180,181]. Unlike SARS-CoV, the processing of SARS-CoV-2 S protein involves the furin enzyme due to the presence of furin cleavage sites in SARS-CoV-2 S protein that are absent in SARS-CoV S protein [182,183]. The uniqueness of the SARS-CoV-2 RBD amino acid sequence and the presence of furin-cleavage sites may explain the rapid spread of SARS-CoV-2 and may be useful in the design of specific anti-SARS-CoV-2 drugs.

**Figure 7:**
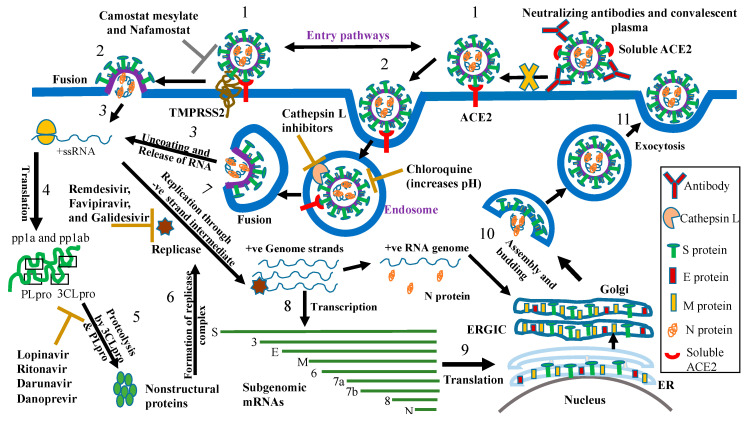
Potential therapeutics targeting different steps of the SARS-CoV-2 life cycle. The steps of the SARS-CoV-2 life cycle are (1) attachment to angiotensin-converting enzyme 2 (ACE2) on target cells, (2) entering the cell by endocytosis (cathepsin L-mediated cleavage of S protein) or through the plasma membrane (TMPRSS2-mediated cleavage of S protein), (3) uncoating and release of viral RNA, (4) translation of viral RNA 5’ end (ORF1a and ORF1b), (5) proteolysis of pp1a and pp1ab by 3CLpro and PLpro into nonstructural proteins, (6) formation of replicase complex, (7) replication of viral RNA, (8) transcription of viral genome into subgenomic mRNAs, (9) translation of subgenomic mRNAs into structural and nonstructural proteins, (10) assembly and budding of new viral particles through Golgi apparatus, and (11) exocytosis and exit of new viral particles out of the cell. Antivirals against SARS-CoV-2 include: entry inhibitors such as convalescent plasma and monoclonal antibodies (e.g., 47D11, HA001, B38, H4, and CR3022), chloroquine, camostat mesylate and nafamostat, cathepsin L inhibitors, and soluble ACE2; 3CLpro inhibitors such as HIV protease inhibitors (lopinavir, ritonavir, and darunavir) and the hepatitis C virus (HCV) protease inhibitor danoprevir; and RdRp inhibitors such as remdesivir, favipiravir, and galidesivir. ER; endoplasmic reticulum, ERGIC; endoplasmic reticulum–Golgi intermediate compartment, and ORF; open reading frame.

Since its emergence in December 2019, many research groups have been working to develop antibodies and small molecules as antivirals for SARS-CoV-2. Experience from SARS-CoV outbreak has provided much guidance in efforts to identify an effective antiviral against SARS-CoV-2. These efforts involve the repurposing of drugs that are either antivirals against viruses such as SARS-CoV, Ebola, HIV, and HCV, or FDA-approved drugs that are approved to treat other health conditions (Table 2). Below are the different agents that have been tested either in vitro or in clinical trials against SARS-CoV-2. These SARS-CoV-2 antivirals are further illustrated in Figure 7 with a clear demonstration of their targets, which are either viral or cellular.

## 5. SARS-CoV-2 Antivirals

### 5.1. Entry Inhibitors (Antibodies and Other Antivirals)

#### 5.1.1. Antibodies

Antibodies against SARS-CoV-2 have been detected around 1–2 weeks after disease onset [184,185]. Polyclonal rabbit anti-SARS-CoV S1 antibodies inhibited the entry of SARS-CoV S-pseudotyped viruses into 293/hACE2 cells but not that of SARS-CoV-2 S-pseudotyped viruses [186]. This suggests that SARS-CoV S1-specific neutralizing antibodies do not cross-react with SARS-CoV-2 S protein. In line with previous findings, convalescent plasma from one SARS-CoV patient neutralized SARS-CoV S-pseudotyped viruses, whereas it had modest inhibitory effect on entry of SARS-CoV-2 S pseudoviruses into 293/hACE2 cells [186]. On the other hand, five samples of convalescent plasma obtained from COVID-19 patients strongly inhibited SARS-CoV-2 S pseudoviral entry with no effect on SARS-CoV S pseudoviral entry. Another study showed that five COVID-19 patients’ sera neutralized live SARS-CoV-2 infection of Vero E6 cells [170]. This suggests that anti-SARS-CoV-2 S-neutralizing antibodies are present in the sera of recovered COVID-19 patients and could be used to treat SARS-CoV-2 active cases. Convalescent plasma has been used to treat COVID-19 patients [187]; however, data on the benefit of this approach are limited. Since convalescent plasma from SARS-CoV patients showed a modest inhibition of SARS-CoV-2 entry [186], neutralizing monoclonal antibodies specifically developed against SARS-CoV-2 S protein could be effective in neutralizing SAR-CoV-2 infections and treating COVID-19 patients. A SARS-CoV S-specific human monoclonal antibody, CR3022, was described in one study as an antibody that strongly binds to SARS-CoV-2 RBD [188]. We believe that CR3022 could be developed to be used as neutralizing antibody for SARS-CoV-2. Another human monoclonal antibody (47D11) was recently developed after its isolation from supernatants of SARS-CoV S hybridomas that were derived from immunized transgenic H2L2 mice [189]. The 47D11 antibody binds a conserved epitope in the RBD of SARS-CoV and SARS-CoV-2. Furthermore, 47D11 neutralized both live SARS-CoV and SARS-CoV-2 infection of Vero E6 cells. However, since very few cross-reacting anti-SARS-CoV S antibodies could be identified due to differences between the two S proteins [7], it is better to develop and identify neutralizing monoclonal antibodies that are specifically targeted to SARS-CoV-2 S protein.

Recently, a human monoclonal antibody, HA001, was described [190]. HA001 neutralized SARS-CoV-2 but not SARS-CoV, by binding to RBD of its S protein. Another group identified anti-SARS-CoV-2 human monoclonal antibodies from a convalescent patient. Two of these antibodies, B38 and H4, recognized different epitopes on SARS-CoV-2 RBD and competed with ACE2 for binding to RBD [191]. When tested for in vivo efficacy in hACE2 transgenic mice, B38 and H4 protected mice from severe SARS-CoV-2-associated pathology and reduced viral titers in lungs. The previous studies indicate that specific anti-SARS-CoV-2 neutralizing monoclonal antibodies are within reach and could be further developed and approved to treat COVID-19 in the near future.

#### 5.1.2. Soluble Recombinant ACE2

Since ACE2 was identified as the receptor for SARS-CoV-2 and soluble ACE2 inhibited SARS-CoV S pseudoviral entry into target cells [61,182], it has been suggested that soluble ACE2 would block SARS-CoV-2 entry into target cells. Recently, a study published in *Cell* showed that human recombinant soluble ACE2 (hrsACE2) inhibited SARS-CoV-2 entry into Vero cells, engineered human blood vessel organoids, and human kidney organoids [192]. The findings of the previous study indicate that hrsACE2 can may be effective in treating COVID-19 patients.

#### 5.1.3. Inhibitors of Endosomal Acidification, Cathepsin L, and TMPRSS2

Chloroquine and hydroxychloroquine inhibited live SARS-CoV-2 entry into Vero E6 cells at a micromolar concentration range [193,194]. The in vivo activities of chloroquine and hydroxychloroquine either alone or in combination with azithromycin are currently being tested in clinical trials (ChiCTR2000029609, NCT04261517, NCT04333628, NCT04341727, NCT04349371, NCT04321278, and NCT04303507). Chloroquine and hydroxychloroquine have been used in treatment protocols in China and other countries based on preliminary results which showed their ability to limit viral replication and ameliorate symptoms in COVID-19 patients [195,196,197]. Hydroxychloroquine plus azithromycin was more effective in reducing viral load in COVID-19 patients compared to hydroxychloroquine alone [198]. Another study showed no significant difference in mortality in patient groups treated with hydroxychloroquine, azithromycin, or hydroxychloroquine plus azithromycin compared to the untreated control group [199]. Therefore, more data are needed from clinical trials in order to confirm the efficacy and safety of chloroquine and hydroxychloroquine in the treatment of COVID-19.

TMPRSS2 and cathepsin L inhibitors blocked entry of SARS-CoV-2 into target cells [182]. In one study published in *Cell*, camostat mesylate (TMPRSS2 inhibitor) and E-64D (cathepsin L inhibitor) blocked SARS-CoV-2 entry into Caco-2 cells and 293T cells, respectively [182]. Nafamostat (another TMPRSS2 inhibitor, Figure 2K) inhibited SARS-CoV-2 entry in Vero E6 cells and lung cells [200]. Camostat mesylate is currently being tested in clinical trials (NCT04321096 and NCT04338906).

#### 5.1.4. Fusion Inhibitors

Umifenovir (Figure 6B) is an approved anti-influenza drug that is used in Russia and China [201]. It inhibits fusion of influenza viral envelope with host cell membranes and it has an immune-enhancing effect [201]. The drug will be tested for its antiviral activity against SARS-CoV-2 in phase 4 clinical trials (NCT04260594, NCT04254874, and NCT04255017).

### 5.2. Replication and Transcription Inhibitors

SARS-CoV-2 uses a replication complex to replicate its _+_ssRNA genome and generate subgenomic viral mRNA that are translated into viral proteins [176]. The replication complex consists of RdRp plus other nonstructural proteins [176]. Remdesivir, an RdRp inhibitor that was previously developed for Ebola, blocked replication of SARS-CoV-2 [193]. Remdesivir effectively inhibited replication of SARS-CoV-2 in Vero E6 cells at a concentration of less than 4 μM [193,202]. Remdesivir is being evaluated for its anti-SARS-CoV-2 activity in phase III clinical trials (NCT04292730, and NCT04292899). Initial results showed that the drug shortened the recovery time to 11 days compared to 15 days in patients who received placebo [202]. On 1 May 2020, FDA announced an “emergency use authorization” (EUA) for remdesivir to treat COVID-19 patients [202]. However, more controlled clinical trials are needed to confirm the efficacy and safety of the drug. Other RdRp blockers such as the anti-HCV nucleoside analogs sofosbuvir and ribavirin were suggested as inhibitors for SARS-CoV-2 based on SARS-CoV-2 RdRp modeling and docking studies [203].

Favipiravir (Figure 4J) is another nucleoside analog that inhibits RdRp and is approved for influenza treatment in Japan [204]. Favipiravir inhibited replication of other viruses such as Ebola, yellow fever, and norovirus [205]. When tested for antiviral activity against SARS-CoV-2, it showed modest inhibition for viral replication in Vero E6 cells [193]. Favipiravir has been used by China to treat COVID-19 patients. In a clinical trial conducted in China, favipiravir reduced viral load and improved clinical outcome with a very few adverse effects [206]. Favipiravir plus hydroxychloroquine, azithromycin, or interferon alpha are being tested in other clinical trials for the treatment of COVID-19 (ChiCTR2000029600 and NCT04411433). Favipiravir combined with baloxavir marboxil (inhibitor of cap-snatching endonuclease activity of the influenza virus polymerase complex) is being tested in another clinical trial for its efficacy against SARS-CoV-2 (ChiCTR2000029544). Penciclovir is another anti-HSV nucleoside analog that showed modest in vitro inhibition of SARS-CoV-2 [193]. Galidesivir, also known as BCX4430 and immucillin-A, is a RdRp inhibitor that showed antiviral activity against SARS-CoV, Ebola, Marburg, and paramyxoviruses [107]. It is currently tested in a clinical trial for its safety and antiviral activity in COVID-19 patients (NCT03891420).

### 5.3. Protease Inhibitors

HIV-1 protease inhibitors such as lopinavir/ritonavir and nelfinavir showed antiviral activity against SARS-CoV. When administered to a 54-year-old male Korean COVID-19 patient, lopinavir/ritonavir decreased viral loads and improved patient condition [207]. Lopinavir and ritonavir are being tested in clinical trials for their efficacy against SARS-CoV-2 (NCT04251871, NCT04261907, NCT04286503, NCT04295551, NCT04276688, NCT04276688, NCT04255017, and ChiCTR2000029539). A completed clinical trial indicated that lopinavir/ritonavir treatment was not effective in COVID-19 patients and did not reduce viral loads compared to controls who received standard supportive care [208]. Serious complications due to COVID-19 were reduced in the treatment group, but the medication caused side effects such as nausea, diarrhea, and hepatotoxicity. Results from other trials are required to fully evaluate the efficacy of lopinavir/ritonavir.

Ritonavir is also being evaluated in a phase 3 clinical trial in combination with the influenza neuraminidase inhibitor oseltamivir (NCT04261270) and in a phase 4 clinical trial in combination with the HCV protease inhibitor danoprevir (Figure 3L) (NCT04345276). Another HIV-1 protease inhibitor, darunavir (Figure 3M), is being tested in a phase 3 clinical trial in China for its activity in the treatment of COVID-19 patients (NCT04252274).

Using molecular modeling of SARS-CoV-2 3CLpro, the anti-HCV NS5A drugs ledipasvir or velpatasvir were identified as promising anti-SARS-CoV-2 candidates [209]. The anti-HCV drug combinations Epclusa (velpatasvir/sofosbuvir) and Harvoni (ledipasvir/sofosbuvir), were also suggested as potential antivirals for SARS-CoV-2 [209].

### 5.4. Miscellaneous Agents

Interferon alpha and beta showed efficacy against SARS-CoV in vitro and in vivo and are approved treatments for other viruses such as HCV and HBV [152,153,154,210]. Based on its broad-spectrum activity as an innate immune system antiviral agent, a clinical trial using the anti-HCV approved combination of pegylated interferon plus ribavirin was initiated to test its efficacy against SARS-CoV-2 (ChiCTR2000029387). Another trial will test the efficacy of interferon alpha combined with lopinavir/ritonavir (NCT04251871). Nitazoxanide (Figure 6C), which is used as an antidiarrheal agent, has been shown to inhibit SARS-CoV-2 replication in Vero E6 cells [193]. The drug works by enhancing host innate interferon responses.

The COVID-19 disease is characterized by dysregulated immune response and excessive production of proinflammatory cytokines [175,211]. Ruxolitinib (Figure 6D) is a small molecule which inhibits JAK1/2 signaling molecules involved in cytokine signaling [212]. It is being tested in clinical trials for its ability to downregulate the immune response and limit the tissue damage that results from immune overactivation by SARS-CoV-2 (NCT04334044). Ruxolitinib is tested in another clinical trial in combination with simvastatin (NCT04348695). Baricitinib (Figure 6E) is another JAK1/2 inhibitor and also an AP2-associated protein kinase 1 (AAK1) inhibitor that has been suggested as a potential drug for SARS-CoV-2 [213]. As an inhibitor for JAK1/2 and AKK1, Baricitinib downregulates inflammation and may block viral entry. Several clinical trials are underway to confirm efficacy in COVID-19 patients (NCT04373044, NCT04401579, NCT04321993, and NCT04345289). The antibody eculizumab, which is used to treat disorders such as paroxysmal nocturnal hemoglobinuria and atypical hemolytic uremic syndrome [214,215], is currently being tested in clinical trials in COVID-19 patients (NCT04355494 and NCT04288713). Eculizumab binds to the complement component C5 and inhibits its cleavage by C5 convertase into C5a and C5b [216]. C5a is an inflammatory complement fragment while C5b activates other complement components which leads to formation of membrane attack complex which damages host cells [217]. Therefore, eculizumab may have protective effects against the inflammation, pneumonia, and lung injury that are associated with COVID-19. Tocilizumab and sarilumab are monoclonal antibodies that block interleukin-6 receptor (IL-6R) and are used for the management of RA [218,219]. Blocking of IL-6R leads to downregulation of the overactive immune responses that are observed in autoimmune diseases such as RA [218]. These antibodies are currently being tested in clinical trials (NCT04346355, NCT04317092, NCT04320615, NCT04306705, NCT04332094, NCT04315298, NCT04324073, and NCT04332913) for the management of COVID-19 since COVID-19 is also characterized by an excessive immune activation that leads to tissue damage. Another clinical trial is combining tocilizumab with favipiravir for the management of SARS-CoV-2 (NCT04310228). Siltuximab is an anti-IL-6 chimeric monoclonal antibody that has been approved for treatment of idiopathic multicentric Castleman disease and has been investigated for use in multiple types of cancer [220,221]. Siltuximab is currently being tested in clinical trials for efficacy and safety in COVID-19 patients (NCT04330638 and NCT04329650).

It is believed that the usage of the ACE2 receptor by SARS-CoV-2 leads to its downregulation [222]. Since ACE2 converts angiotensin II (AngII) into angiotensin I (AngI), its downregulation could lead to accumulation of AngII. Elevated levels of AngII cause an increase in blood pressure and other undesirable effects such as increased pulmonary vascular permeability and lung tissue damage [222,223]. Therefore, use of angiotensin II receptor blockers (ARBs) such as valsartan (Figure 6F) and telmisartan (Figure 6G) may have a protective effect in COVID-19 patients [223]. Clinical trials testing the beneficial effect of ARBs in COVID-19 patients are currently underway (NCT04355936 and NCT04335786).

## 6. Conclusions

SARS-CoV and SARS-CoV-2 represent two HCoVs with high fatality rates and no approved antivirals to date. Their genome sequence identity (79%) could be very helpful in developing broad-spectrum monoclonal antibodies. Since both viruses utilize same receptor (ACE2), entry pathways, and replication strategies, antivirals that have shown efficacy against SARS-CoV are currently being tested in clinical trials and could be further developed and approved to combat SARS-CoV-2. Remdesivir and favipiravir are the most promising antiviral drugs that have been tested in clinical trials so far. However, a recent study analyzed 220 SARS-CoV-2 genomic sequences and reported mutations in different viral proteins including RdRp [224]. It is important to study these mutations to identify any drug-resistant viral variants. This could lead to characterization of more effective therapeutics for SARS-CoV-2.

## Figures and Tables

**Table 1:** Severe Acute Respiratory Syndrome-coronavirus (SARS-CoV) antivirals: Targets and mechanisms of action.

Target of Antivirals	Example/s	Mechanism of Action
Viral Entry		
**• Spike (S) protein**	Monoclonal Antibodies such as S230.15, m396, S109.8 S227.14, S230.15, 80R scFv, CR3022 CR3014, 33G4 35B5, 30F9, 4D4, IF8, 5E9, and B1 scFv	Most of the antibodies bind to S1 domain of SARS-CoV S protein and block its binding to ACE2 on target cells. Antibodies, such as 4D4, bind to regions other than RBD and block a post-attachment step in viral entry. Others (1F8, 5E9, and B1) bind to S2 domain and inhibit viral envelope-cell membrane fusion
	Convalescent plasma	Contains neutralizing antibodies that bind to different regions on SARS-CoV S protein and inhibit viral entry by different mechanisms
	Lectins: Griffithsin (a lectin isolated from the red alga *Griffithsia spp.*) and Urtica dioica agglutinin (UDA) (a lectin isolated from *Urtica dioca*)	Bind to SARS-CoV S protein and inhibit binding of SARS-CoV to ACE2
	Synthetic peptides derived from ACE2 and soluble ACE2 ectodomain	Bind to SARS-CoV S protein and inhibit attachment to target cells
	Synthetic peptides derived from HR1 or HR2 regions of S2 domain e.g. CP-1 peptide.	Bind to S2 domain and block fusion of viral envelope with cell membrane
	Imatinib (Abl kinase inhibitor)	Blocks fusion of viral envelope with cell membrane
	Chinese herbal medicine molecules tetra-O-galloyl-beta-D-glucose (TGG) and luteolin	Bind to S2 domain and inhibit fusion
	Emodin (a plant anthraquinone)	Binds to SARS-CoV S protein and blocks its interaction with ACE2
**• Angiotensin-converting enzyme 2** **(ACE2)**	N-(2-aminoethyl)-1-aziridineethanamine (NAAE)	Inhibits ACE2 activity and blocks fusion
**• Clathrin-mediated endocytosis**	Chlorpromazine	Inhibits viral entry by blocking clathrin-mediated endocytosis of viral particles
**• Endosomes**	Chloroquine and P9 peptide	Raise the pH of the endosome and thus inhibit viral entry by blocking cathepsin L-mediated cleavage of S protein
**• Cathepsin L**	Small molecules such as E-64D and 5705213	Inhibit cathepsin L and thus inhibits cathepsin L-mediated cleavage of S protein
**• Transmembrane Serine Protease 2** **(TMPRSS2)**	Camostat mesylate	Blocks TMPRSS2 and thus inhibits processing of SARS-CoV S protein and viral entry at plasma membrane
**RNA-dependent RNA polymerase** **(RdRp)**	Nucleoside analogs: β-D-N4-hydroxycitidine, galidesivir also known as BCX4430 or immucillin-A, and remdesivir	Inhibit RdRp leading to premature termination of RNA synthesis
	Nucleoside analogs: 6-azauridine and pyrazofurin	Inhibit orotidine monophosphate decarboxylase which leads to depletion of pyrimidine nucleosides pool
	Nucleoside analog: ribavirin	Ribavirin gets incorporated into the newly synthesized RNA chain resulting in viral lethal mutations. It is also believed to inhibit cellular inosine monophosphate dehydrogenase resulting in depletion of intracellular GTP which leads to inhibition of mRNA cap synthesis
**Viral helicase**	Bananins	Inhibit ATPase activity of viral helicase and thus blocks viral replication
	Bismuth complexes	Inhibit ATPase and unwinding activities of viral helicase
	Falvonoids such as myricetin and scutellarein	Inhibit ATPase activity of viral helicase.
	Small molecule SSYA10-001	Inhibits unwinding activity of viral helicase
**Viral proteases**		
**• Picornavirus 3-chymotrypsin-like protease (3CLpro)**	HIV-1 protease inhibitors; lopinavir/ritonavir and nelfinavir	One of the mechanisms is inhibition of 3CLpro activity which leads to inhibition of processing of SARS-CoV polyproteins and blockage of viral replication. Other mechanisms involve inhibition of the cellular enzymes; calpains
	Cinanserin (a serotonin antagonist), esculetin-4-carboxylic acid ethyl ester (a marine natural product; coumarin derivative), plant-derived compounds such as betulinic acid (sesquiterpenoid) and savinin (lignoid), quinone-methide trit-erpenes isolated from *Tripterygium regelii*, dieckol (a phlorotannin) isolated from brown algae *Ecklonia cava*, alkylated chalcones from *Angelica keiskei*, flavonoids such as herbacetin, rhoifolin and pectolinarin, and pyrazolone compounds	Inhibit 3CLpro and thus inhibit viral replication
**• Papain-like protease 2** **(PLpro)**	Diarylheptanoids from *Alnus japonica* and geranylated flavonoids from *Paulownia* tree, 6-mercaptopurine (6-MP), 6-thioguanine (6-TG), and disulfiram	Inhibit viral replication by blocking PLpro and processing of viral polyproteins
**Cellular proteases:** **Calpain**	Z-Val-Phe-Ala-CHO (calpain inhibitor III) and Val-Leu-CHO (calpain inhibitor VI)	Inhibit calpains and thus inhibit viral replication and virus-induced apoptosis
**Miscellaneous Antivirals**	E, M, N, ORF3a, ORF7a, and ORF7b siRNAs	Inhibit viral replication by inducing the breakdown of viral mRNAs
	Hexamethylene amiloride	Blocks E protein cationic-selective ion channel activity
	Aurintricarboxylic acid	Inhibits SARS-CoV attachment to target cells and inhibits viral replication
	Rimantadine	Blocks E protein cationic-selective ion channel activity.
	Niclosamide	Inhibits replication of SARS-CoV. Mechanism is not well known
	Amiodarone	Inhibits post-entry step in viral life cycle
	K22	Inhibits formation of double membrane vesicles
	dsRNA-activated caspase oligomerizer (DRACO)	Induces apoptosis in cells that are infected with SARS-CoV by recognizing viral dsRNA intermediates using its viral dsRNA-binding domain
	NO donors: 2,2′-(hydroxynitrosihydrazino)bis-ethamine (DETA NONOate) and S-nitroso-N-acetylpenicillamine (SNAP)	Dissociate to produce NO which is antiviral
	Cyclosporine A and its analog alisporivir	Form a complex with cyclophilins which leads to inhibition of viral replication. Also, the complex with cyclophilins inhibits calcineurin which blocks T cell activation and subsequent T cell-mediated tissue damage
	Geldanamycin (an antibiotic)	Binds to and inhibits heat shock protein 90 (Hsp90) which inhibits SARS-CoV replication
	Interferon alpha and beta	Viral RNA degradation, inhibition of protein synthesis, and viral assembly
	Glycyrrhizin (triterpenoid saponin glycoside from licorice root)	Inhibits adsorption of viral particles on target cells and induces production of NO. Other antiviral mechanisms need to be explored

**Table 2:** SARS-CoV-2 antivirals: Targets and mechanisms of action.

Target of Antivirals	Example/s	Mechanism of Action
Viral entry		
**• S protein**	Convalescent plasma	Contains neutralizing antibodies that block binding of SARS-CoV-2 S protein to ACE2
	Monoclonal antibodies (47D11, HA001, B38, H4, and CR3022)	Neutralize SARS-CoV-2 by binding to S protein and blocking attachment to ACE2
	Umifenovir (is currently tested in clinical trials against SARS-CoV-2)	An anti-influenza drug that inhibits fusion of viral envelope with cellular membranes
	Soluble ACE2	Binds to SARS-CoV-2 S protein and inhibits attachment to target cells
**• Endosomes**	Chloroquine and hydroxychloroquine (currently tested in clinical trials)	Raise the pH of the endosomes and thus inhibit viral entry by blocking cathepsin L-mediated cleavage of S protein
**• Cathepsin L**	Small molecule E-64D and 5705213	Inhibit cathepsin L and thus inhibits cathepsin L-mediated cleavage of S protein
**• TMPRSS2**	Camostat mesylate (is currently evaluated in a clinical trial) and nafamostat meslylate	Block processing of SARS-CoV-2 S protein by TMPRSS2 and thus inhibits viral entry at plasma membrane
**RdRp**	Remdesivir (is currently evaluated in clinical trials), penciclovir, favipiravir (favipiravir plus interferon alpha and favipiravir plus baloxavir marboxil are currently tested in clinical trials), and galidesivir (is currently tested in clinical trials)	Inhibit RdRp leading to premature termination of RNA synthesis
**Viral Proteases** **• 3CLpro**	HIV protease inhibitors: lopinavir, ritonavir, darunavir and lopinavir/ritonavir combination (are currently tested in clinical trials)	Inhibition of 3CLpro activity which leads to inhibition of processing of SARS-CoV-2 polyproteins and blockage of viral replication
	HCV protease inhibitor: danoprevir (is currently tested in a clinical trial combined with ritonavir)	Inhibition of 3CLpro activity
**Miscellaneous Agents**	Interferon alpha and beta (is currently tested in a clinical trial combined with ribavirin and in another trial combined with lopinavir/ritonavir)	Viral RNA degradation, inhibition of protein synthesis, and viral assembly
	Nitazoxanide	Enhances host innate interferon responses
	Ruxolitinib (is currently tested in clinical trials alone or combined with Simvastatin) and baricitinib	Inhibit JAK1/2 signaling and thus downregulate immune responses that are responsible for tissue damage. Barcitinib may block viral entry
	Eculizumab antibody (is currently tested in clinical trials)	Downregulates inflammation by binding to the complement component C5 and inhibits its cleavage by C5 convertase into C5a and C5b
	Tocilizumab (is currently tested in clinical trials alone or combined with favipiravir) and sarilumab	Monoclonal antibodies that block IL-6R and thus downregulate inflammation that is associated with COVID-19 disease
	Siltuximab (is currently tested in clinical trials)	An anti-IL-6 antibody that ameliorates inflammation and downregulates damaging immune responses.
	Valsartan and telmisartan (are currently tested in clinical trials)	Are angiotensin II receptor blockers that protect against harmful effects of angiotensin II in COVID-19 patients
